# Construction of a Humanized PBMC-PDX Model to Study the Efficacy of a Bacterial Marker in Lung Cancer Immunotherapy

**DOI:** 10.1155/2022/1479246

**Published:** 2022-08-28

**Authors:** Chengwei Wu, Xinning Wang, Haitao Shang, Hong Wei

**Affiliations:** Precision Medicine Institute, The First Affiliated Hospital, Sun Yat-sen University, Guangzhou 510080, China

## Abstract

Commensal microbiome is a key factor of lung cancer immunotherapy efficacy. Elucidating the role of specific strains as bacterial markers in immunotherapy has drawn great attention from the academia. At present, most preclinical studies about the relationship between bacterial markers and immunotherapy rely on the syngeneic mouse models. However, mice differ greatly from humans in immune system and tumor characteristics. In this study, humanized mouse models based on peripheral blood mononuclear cells (PBMCs) immune reconstitution and lung cancer cell line-derived xenograft (CDX) or patient-derived xenograft (PDX) were constructed. The PBMC-PDX model was shown to be superior to the PBMC-CDX model in preserving tumor heterogeneity and construction time-saving. Through optimizing the experimental process, the time it took for humanized models to evaluate the effect of cancer treatment was reduced to 42 days. Next, by utilizing PBMC-PDX mice treated with antibiotics (ATB), the role of *Bifidobacterium longum* in lung cancer immunotherapy was studied. It was found that although both *Bifidobacterium longum* and immunotherapy drug pembrolizumab alone showed suppressing tumor growth, the efficacy of pembrolizumab was attenuated when administrated to mice colonized with *Bifidobacterium longum*. Further exploration revealed that *Bifidobacterium longum* caused significant changes in the proportion of human CD45^+^ cells in the PBMC-PDX model. The PBMC-PDX model has the potential to be applied as an efficient platform to support evaluation of bacterial markers in immunotherapy research and facilitate development of precision medicine targeting human commensal bacteria.

## 1. Introduction

Worldwide, lung cancer remains the highest cause of cancer-related mortality [1]. And, 80% of lung cancer deaths are from non-small cell lung cancer (NSCLC) [2]. Immunotherapy has been shown to have good survival benefits with advanced NSCLC [3]. Pembrolizumab showed superior efficacy, when patients express PD-L1 in at least 50% of tumor cells, and was better tolerated as first-line therapy compared to platinum doublet chemotherapy [4]. Although breaking the PD-1/PD-L1 axis has significant therapeutic effect and contributes to prolongation of patient survival, the response rate of NSCLC patients is only about 20% [5]. It may be related to intestinal microbiota.

The growing evidence support that the microbiome has a regulatory role in cancer immunotherapy responses [6–8]. The latest research shows that intestinal symbiotic bacteria influence the efficacy of anti-PD-1 antibodies in melanoma and lung cancer and *Bifidobacterium longum* is enriched in responders [9, 10]. A large number of mouse experiments have been carried out to study the regulatory effect of intestinal microbiota on cancer immunotherapy [11]. However, most of the current studies on bacterial markers rely on mouse immune system and mouse tumor [12–14], which cannot simulate the real situation of bacterial flora and cancer immunotherapy in human body. Therefore, it is urgent to develop suitable humanized models.

Cell line-derived xenograft (CDX) mice are often used to test the antitumor drug [15, 16]. However, CDX model constructed solely from tumor cell lines cannot simulate the complex human tumor environment [17]. Patient-derived xenograft (PDX) mice have been identified as better preclinical models [18]. The PDX model retains most of the tumor tissue components, which can be better used to predict the sensitivity and resistance of drug [19, 20]. In addition, these two types are often used in humanized immune system of mice [21–24]. Compared with the humanized mice using CD34^+^ hematopoietic stem cells (HSCs), the humanized model constructed by peripheral blood mononuclear cells (PBMCs) takes less time, but graft versus-host disease (GvHD) occurs [25, 26]. At present, studies on bacterial markers and immunotherapy mostly adopt sterile mouse models and antibiotics (ATB) treated mice [27–29]. However, weighing the acquisition cost [30], mouse models treated with ATB are relatively more economical.

Here, we established a PBMC-PDX model that exhibited advantages over its CDX counterpart. With respect to the utilization of this model in immunotherapy research, the experimental process was optimized. The efficacy of *Bifidobacterium longum* in lung cancer immunotherapy was studied in virtue of this PBMC-PDX model, which could be further applied to elucidate the molecular mechanisms in bacterial marker-mediated immune responses.

## 2. Materials and Methods

### 2.1. Mice

In the Laboratory Animal Center of Sun Yat-sen University, all animal experiments were conducted. All executed procedures were in compliance with the Animal Welfare Committee of the Laboratory Animal Center. NOD-scid-IL2Rg−/− (NPI) mice were provided by BEIJING IDMO CO., LTD. The specific pathogen-free (SPF) cages were used to raise mice. The institutional animal care and use committee (IACUC) approves animal protocol.

### 2.2. Cell Line

A549 (non-small cell lung carcinoma cell line of human) was purchased from Procell Life Science & Technology Co., Ltd., and the RPMI-1640 Complete Medium (10%FBS; Gibco) was used for culture. When the cells reached 80% confluence, they were passed on.

### 2.3. PBMCs Immune Reconstitution

PBMCs were purchased from AllCells and processed according to the company's operating manual. Mice intravenously transplanted with 1 × 10^7^ human PBMCs.

Pembrolizumab was purchased from Target Molecule Corp. and injected intraperitoneally into mice (250 *μ*g/mouse) every three days.

### 2.4. CDX Model

Back injection of A549 cells (1 × 10^6^) to NPI mice at 6 to 8 weeks of age was conducted. Mice were injected with pembrolizumab 30 days after injection of A549 cells. Growth of tumor was monitored every three days. The formula (length × width^2^)/2 is used to calculate tumor volume.

### 2.5. PDX Model

The lung tumor tissues of human was provided by BEIJING IDMO CO., LTD. Tissues use were approved by the committee for the ethical review of research involving human subjects at the First Affiliated Hospital of Sun Yat-sen University. The tissue was dissected into pieces two millimeters in diameter and placed in phosphate-buffered saline (PBS) containing antibiotics. Then, these pieces were subcutaneously transplanted into 6-8-week-old NPI mice. Tumor growth was monitored when the tumor volume reached 1200 mm^3^, and they are passed on afterwards.

### 2.6. PBMC-CDX Model

The CDX model was constructed, and then, 30 days later, mice were given PBMCs to construct the PBMC-CDX model. Mice were treated with pembrolizumab on the same day after being given PBMCs.

### 2.7. PBMC-PDX Model

**Figure 1:**
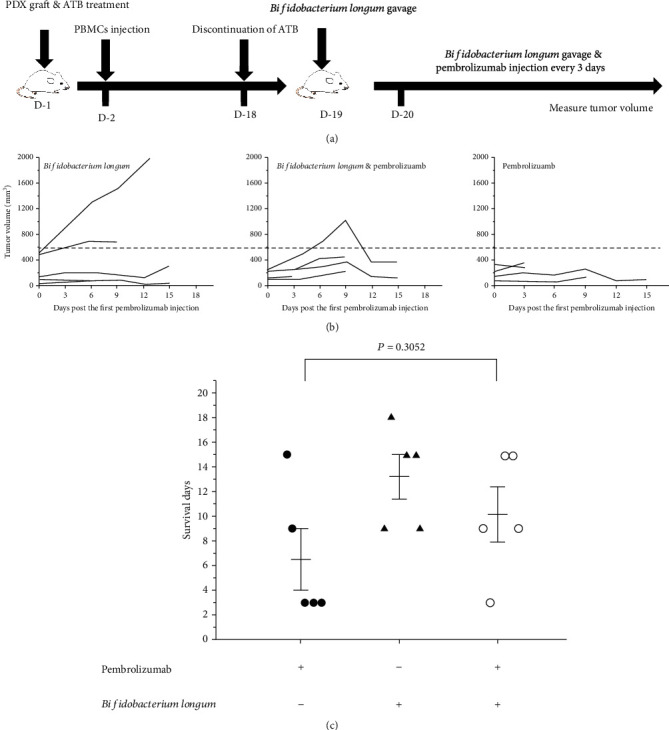
Establishing PBMC-PDX mice and evaluating the effect of *Bifidobacterium longum* and pembrolizumab on the models. (a) The experimental scheme about evaluating the therapeutic effect of *Bifidobacterium longum* and pembrolizumab in PBMCs immune reconstituted PDX mouse models. (b) Tumor growth curve treated with *Bifidobacterium longum* and pembrolizumab; *n* = 5. (c) Death of mice. ^∗^*P* < 0.05, ^∗∗^*P* < 0.01, and ^∗∗∗^*P* < 0.001. Means ± SEM are used in graphs. (c) Two-sample *t*-test. Survival days, *P* value was not significant.

**Figure 2:**
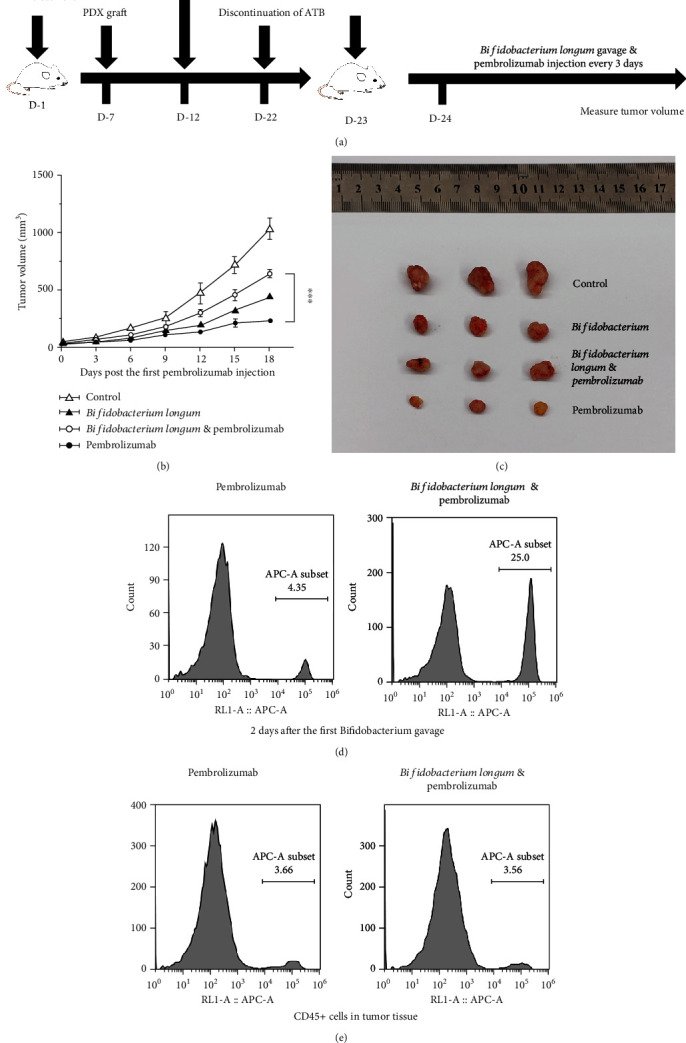
Optimizing the experimental scheme. (a) Optimized experimental scheme. (b) Tumor growth curve treated with *Bifidobacterium longum* and pembrolizumab; *n* for the control, *Bifidobacterium longum*, *Bifidobacterium longum*, pembrolizumab, and pembrolizumab are 5, 3, 5, and 4, respectively. The control group was treated with PBS. (c) Representative tumor pictures. (d) Human CD45^+^ cell in peripheral blood of PBMC immune reconstituted PDX mouse model at the appointed time. (e) Human CD45^+^ cell in tumor tissues. ^∗^*P* < 0.05, ^∗∗^*P* < 0.01, and ^∗∗∗^*P* < 0.001. Means ± SEM are used in graphs. (b) Two-sample *t*-test. The *P* values of curve were significance.

The PDX model was constructed, and then PBMCs were given to construct the PBMC-PDX model at different time points according to different experimental plans, as shown in Figures 1(a) and 2(a).

### 2.8. HE Staining

Tissue blocks were collected, fixed, paraffin embedded, and sectionalized at 4 *μ*m. The slices were dewaxed with xylene and washed with ethanol. Dye with hematoxylin for 5 minutes and rinse with running water. Differentiation with hydrochloric acid ethanol solution was done for 30 s. Soak in tap water for 15 min and eosin solution for 2 min, followed by conventional dehydration, transparent, sheet sealing. Images were acquired using the Leica optical microscope (Leica Biosystems Imaging, USA).

### 2.9. Antibiotics (ATB) Treatment

**Figure 3:**
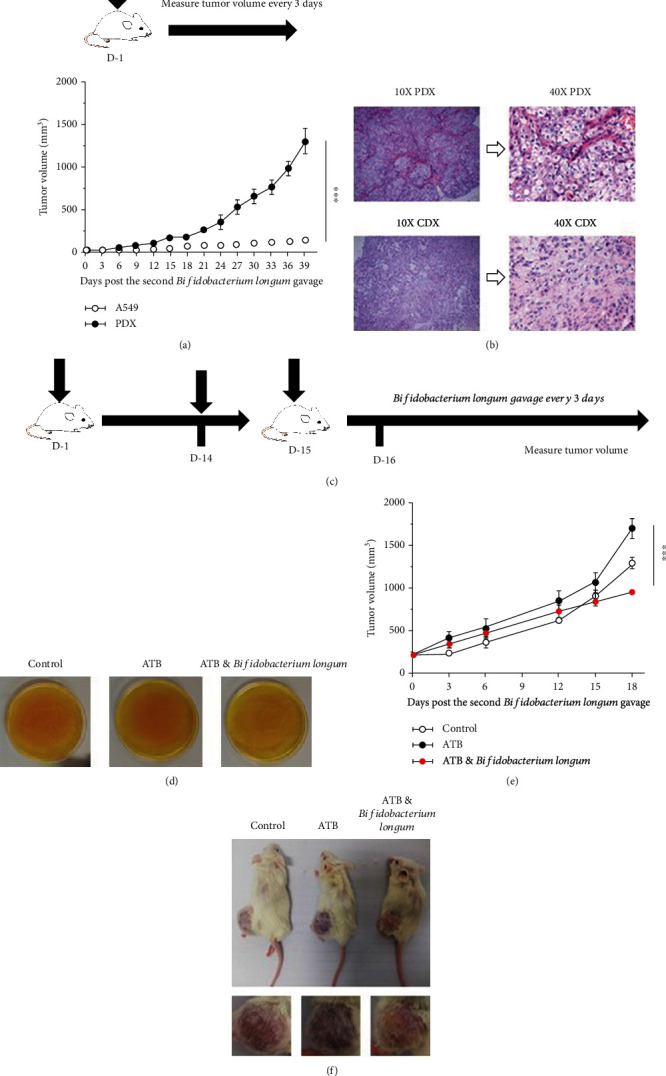
Establishing PDX mice and evaluating the effect of *Bifidobacterium longum* on PDX mouse models. (a) Comparison between the PDX model and CDX model. Tumor curves showed that PDX mouse model tumor growth was significantly faster than CDX mouse model; *n* = 5. (b) Histological morphology of PDX mouse model and CDX mouse model. HE staining was used. Scale bar: 2 mm. (c) Experimental design to evaluate the therapeutic effect of *Bifidobacterium longum* on PDX mouse model. (d) Anaerobic culture of mouse feces on BBL plate medium. The control group was not treated with antibiotics (ATB). (e) Effect of *Bifidobacterium longum* on PDX model. Tumor curves showed that *Bifidobacterium* longum reduced tumor growth; *n* = 5. (f) Representative mouse and tumor pictures. ^∗^*P* < 0.05, ^∗∗^*P* < 0.01, and ^∗∗∗^*P* < 0.001. Means ± SEM are used in graphs. (a) Two-sample *t*-test. (e) One-way ANOVA. The *P* values of curves (a, e) are significance.

Ampicillin (1 mg/ml), colistin (1 mg/ml), streptomycin (5 mg/ml), and vancomycin (0.25 mg/ml) were configured to form ATB cocktail. ATB cocktail was added to the drinking water of mice. The ATB treatment group maintained antibiotic treatment until the end of the experiment (Figure 3(d)).

### 2.10. Bacterial Administration

*Bifidobacterium longum* was purchased from BeNa Culture Collection (BNCC) ORG. CN. with its number BNCC185354. BBL medium was used to continue bacterial culture in an anaerobic environment according to the operating manual provided by the company. *Bifidobacterium longum* was resuspended in PBS at 1 × 10^9^ CFU/ml, and each mouse was given 100 *μ*l by oral gavage.

### 2.11. Fecal Culture

48 hours after discontinuation of antibiotic treatment, 2-3 feces of mice were collected and suspended in 1 ml PBS (Figure 3(d)). The supernatant was cultured in BBL medium in an anaerobic environment.

### 2.12. Flow Cytometry

Tumor tissues were dissected into pieces and placed in PBS including 0.2 mg/ml hyaluronidase V, 0.5 mg/ml collagenase A, and 0.02 mg/ml DNase I. The pieces were digested for 30 min at 37°C. A 70 *μ*m cell strainer (Falcon) was used to filter the suspension. A discontinuous 40% followed by an 80% Percoll® Density Gradient Media (Solarbio) was used for isolation and purification. After collecting peripheral blood, the Red Blood Cell Lysis Buffer (Solarbio) was used to lyse the cells according to the instruction. Leukocyte was collected at 400 r.c.f. for 5 min.

All antibodies were provided by Biolegend. An Invitrogen™ Attune™ NxT device (Thermo Fisher Scientific, USA) was used to perform flow cytometric analysis. Data analysis uses the FlowJo software (v.10.8.1, FlowJo, USA). APC anti-human CD45 (HI30) and FITC anti-human CD3 (HIT3a) were used in these analyses.

### 2.13. Statistical Analysis

Statistical analysis is detailed in the figure legends. Two-sample *t*-test or one-way ANOVA was used to analyze the differences using the Origin9.0 software. Statistically significant *P* value is <0.05.

## 3. Results

### 3.1. Establishing PBMCs Humanized Mice and Evaluating the Effect of Immunotherapy on the Model

**Figure 4:**
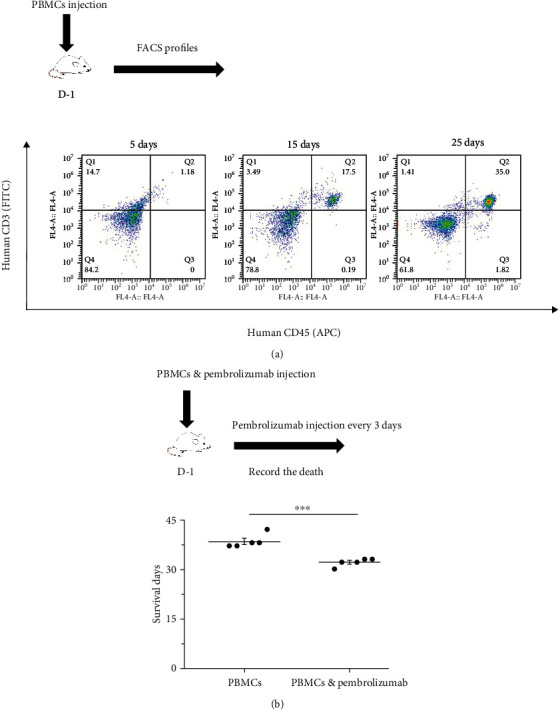
Establishing PBMCs humanized mice and evaluating the effect of immunotherapy on the model. (a) PBMCs humanized mice. FACS profiles of human CD45^+^ and CD3^+^ in NPI mice at the appointed time. (b) Effect of immunotherapy. The survival time decreased after pembrolizumab treatment; *n* = 5. ^∗^*P* < 0.05, ^∗∗^*P* < 0.01, and ^∗∗∗^*P* < 0.001. Means ± SEM are used in graphs. Two-sample *t*-test. Statistics on survival days (b). *P* values are significance.

To detect the immune reconstitution characteristics of PBMCs humanized mice, we only injected mice with PBMCs and monitored the growth of its human CD45^+^ and CD3^+^ cells. Flow cytometry results showed that there were two groups of CD45^+^- and CD3^+^-positive cells or CD45^−^- and CD3^−^-negative cells. Meanwhile, the proportion of double positive cells in peripheral blood gradually increased over time (Figure 4(a)). At the same time, we assessed the impact of immunotherapy on the model. Pembrolizumab was administered after injection of PBMCs. The results showed that pembrolizumab accelerated death in mice (Figure 4(b)). These results indicated that PBMC humanized mice were successfully constructed.

### 3.2. Establishing PBMC-CDX Mice and Evaluating the Effect of Immunotherapy on the Model

**Figure 5:**
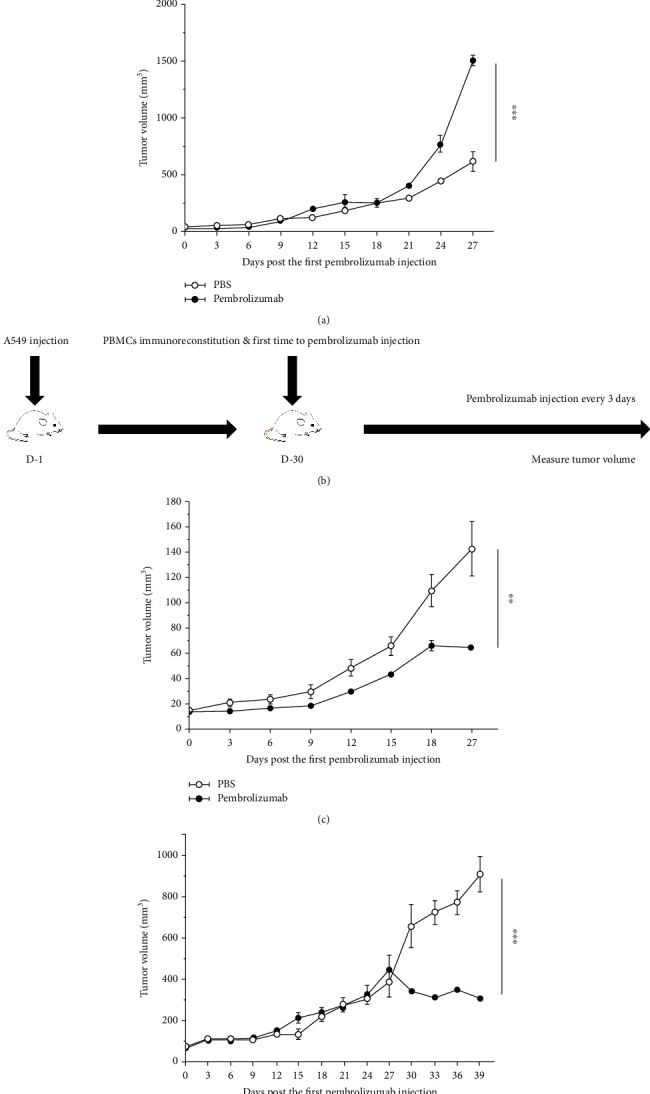
Establishing PBMC-CDX mice and evaluating the effect of immunotherapy on the model. (a) Effect of pembrolizumab on CDX model. Tumor curves showed that pembrolizumab promoted tumor growth in the absence of PBMCs immune reconstitution; *n* = 5. The control group was treated with PBS. (b) The experimental scheme about evaluating the therapeutic effect of pembrolizumab in PBMCs immune reconstituted CDX models. (c) Therapeutic efficacy of pembrolizumab on PBMC-CDX model. Growth curve of tumor showed that pembrolizumab has an antitumor efficacy on PBMC immune reconstituted CDX mouse models; *n* = 5. (d) The injection dose of PBMCs and pembrolizumab was halved. Tumor curves showed that tumor growth was reduced after long-term treatment in PBMCs immune reconstituted CDX mouse models; *n* = 5. ^∗^*P* < 0.05, ^∗∗^*P* < 0.01, and ^∗∗∗^*P* < 0.001. Means ± SEM are used in graphs. Two-sample *t*-test was used. The *P* values of curves (a, c, d) are significance.

Next, we constructed a CDX mouse model of A549 to evaluate the effect of immunotherapy. The results showed that pembrolizumab increased tumor growth at day 27 after 10 treatments (Figure 5(a)). Subsequently, a PBMC-CDX model was constructed to validate the antitumor efficacy of pembrolizumab (Figure 5(b)). In the presence of PBMCs, pembrolizumab slowed tumor growth after 8 treatments (Figure 5(c)). In consideration of the occurrence of GvHD in the PBMCs model (data not shown), we reduced the dose of pembrolizumab and PBMCs to extend the therapeutic time window. The result showed that after 10 treatments with pembrolizumab, there was also a tendency to slow tumor growth (Figure 5(d)). Reducing the dose could prevent mice from dying but weaken the efficacy of tumor treatment and prolong the experimental period.

### 3.3. Establishing PDX Mice and Evaluating the Effect of *Bifidobacterium longum* on PDX Mouse Models

Considering the slow growth of tumor in the CDX model, we decided to replace the CDX with PDX. The tumor growth rate of PDX model was faster than CDX model (Figure 3(a)). HE staining of tumor tissue showed obvious tumor parenchyma and stroma in PDX, but not in CDX (Figure 3(b)). In order to verify the influence of *Bifidobacterium longum* on PDX model, we used antibiotics (ATB) to process PDX model, and the experimental process is shown in Figure 3(c). Fecal culture on BBL medium showed no colony culture in ATB treatment group, but significant bacterial growth in control group and *Bifidobacterium longum* group (Figure 3(d)). At the same time, compared with the control group and ATB treatment group, after 7 times of *Bifidobacterium longum* treatment, the tumor growth rate was slowed down, and obvious inflammatory and purulent reaction occurred at the tumor site (Figures 3(e) and 3(f)). The above results showed that compared with the CDX model, the PDX model was closer to the actual growth situation of tumor in human body, and *Bifidobacterium longum* had antitumor efficacy in the PDX model.

### 3.4. Establishing PBMC-PDX Mice and Evaluating the Effect of *Bifidobacterium longum* and Pembrolizumab on the Models

Given the antitumor effects of *Bifidobacterium longum*, we plan to construct an ATB-treated PBMC-PDX model to evaluate the effect of *Bifidobacterium longum* on immunotherapy. On day 10 after PBMCs inoculation, the proportion of CD45^+^ cells was 4.16% by flow cytometry (data not shown). Given that the PBMC mice were available for approximately 35 days and that immune reconstructive levels reached high levels after 15 days, we began treatment with *Bifidobacterium longum* and pembrolizumab on day 18 after PBMC transplantation, as shown in Figure 1(a). Mice that received *Bifidobacterium longum* and pembrolizumab tended to have faster tumor growth than mice that received pembrolizumab alone (Figure 1(b)). At the same time, with the progress of the experiment, mice died successively. Comparing the death of mice treated with pembrolizumab and *Bifidobacterium longum* and pembrolizumab, *Bifidobacterium longum* may slow down the role of pembrolizumab in promoting GvHD, although there is no statistical difference (Figure 1(c)). Mice died during the study, but these results suggest that *Bifidobacterium longum* and pembrolizumab alone treatment may slow tumor growth, whereas *Bifidobacterium longum* combined with pembrolizumab may not exert further antitumor efficacy.

### 3.5. Optimizing the Experimental Scheme

To avoid mouse death during the trial, PBMC administration was performed 12 days before pembrolizumab injection. Meanwhile, according to the time of tumor growth to 120-180 mm^3^, we set the timing of tumor inoculation on the 17th day before drug administration; the experimental process is shown in Figure 2(a). The results showed that both *Bifidobacterium longum* and pembrolizumab had antitumor efficacy; however, the combination of the two attenuated the antitumor efficacy (Figures 2(b) and 2(c)). These results are basically consistent with the above experimental results.

After two days of *Bifidobacterium longum* gavage, the peripheral blood has an increased proportion of CD45^+^ cells of the *Bifidobacterium longum* and pembrolizumab group compared with the pembrolizumab group (Figure 2(d)). Finally, tumor cells from the mice were collected for flow cytometry. The proportion of infiltrated human CD45^+^ cells in the tumor was no significant difference (Figure 2(e)). These results indicate that the optimized experimental procedure can be used to study the interaction of *Bifidobacterium longum* and pembrolizumab.

## 4. Discussion

In this study, we constructed PBMC-CDX and PBMC-PDX models of lung cancer and analyzed the characteristics of the two. The experimental result showed that pembrolizumab promotes GvHD in the humanized mouse model of PBMCs, which is consistent with related reports [31, 32]. In general, PBMC humanized mice were able to construct human T cells to a great extent, but the presence of GvHD reduced the duration of the experiment, especially after pembrolizumab treatment. Meanwhile, we found that pembrolizumab promoted GvHD in the absence of PBMCs immune reconstitution. This is consistent with reports that anti-PD-1 antibodies promote tumor hyperprogression [33]. These results suggest that immune reconstruction is the basis for evaluating the antitumor efficacy of immunotherapy. Next, we verified that the PBMC-PDX model was closer to the human situation and had a shorter experimental period for subsequent evaluation of a bacterial marker in immunotherapy. Although a PBMC-PDX model has been identified as a model for assessing the anti-PD-1 antibody efficacy, the application of PBMC-PDX model in clarifying the role of *Bifidobacterium longum* in immunotherapy has not been reported. Our study showed that the ATB-treated PBMC-PDX model has an ability to evaluate the antitumor efficacy of *Bifidobacterium longum* and pembrolizumab.

In cancer research, CDX and PDX models are common models. Recently, it has been reported that PDX has various advantages over CDX in preserving tumor heterogeneity and microenvironment [34]. Our results show that the PDX model is more similar in characteristics of human tumor. Meanwhile, given the antitumor ability of *Bifidobacterium longum* in PDX model without immune reconstitution itself may be a potential model to evaluate the antitumor effect of bacterium.

Although it has been reported, *Bifidobacterium* is beneficial to pembrolizumab treatment in lung cancer [9]. A study based on the syngeneic mouse model reported that even if the bacterium has antitumor effect, it will not promote immunotherapy, and only specific strains are useful [35]. Our results showed that in the ATB-treated PBMC-PDX model, although *Bifidobacterium longum* played an antitumor role, it showed an antagonistic effect against pembrolizumab treatment. Flow cytometry results of peripheral blood showed that *Bifidobacterium longum* treatment induced significant changes in proportion of CD45^+^ cell.

In summary, we have constructed a PBMC-PDX model for evaluating the role of *Bifidobacterium longum* in lung cancer immunotherapy. The establishment of the model and the optimization of the experiment process were performed. This model may provide an alternative and suitable experimental platform for the study of bacterial markers in cancer immunity and help to further discover the underlying mechanism to guide individualized immunotherapy in the foreseeable future.

## Data Availability

Data supporting the findings of this study are available on request from the corresponding author.
